# *CYP2C19*-rs4986893 confers risk to major depressive disorder and bipolar disorder in the Han Chinese population whereas *ABCB1*-rs1045642 acts as a protective factor

**DOI:** 10.1186/s12888-022-04514-w

**Published:** 2023-01-25

**Authors:** Ting Zhang, Qingmin Rao, Kangguang Lin, Yongyin He, Jintai Cai, Mengxin Yang, Ying Xu, Le Hou, Yulong Lin, Haiying Liu

**Affiliations:** 1 Clinical Laboratory, The Affiliated Brain Hospital of Guangzhou Medical University, Guangzhou, China; 2 Affective Disorder Department, The Affiliated Brain Hospital of Guangzhou Medical University, Guangzhou, China; 3 Department of Neurology, The Affiliated Brain Hospital of Guangzhou Medical University, Guangzhou, China

**Keywords:** Major depressive disorder, Bipolar disorder, Genetics, Risk factor, Susceptibility

## Abstract

**Background:**

Genetic risks may predispose individuals to major mood disorders differently. This study investigated the gene polymorphisms of previously reported candidate genes for major depressive disorder (MDD) and bipolar disorder (BPD) in the Han Chinese population.

**Methods:**

Twenty loci of 13 candidate genes were detected by MALDI-TOF mass spectrometry in 439 patients with MDD, 600 patients with BPD, and 464 healthy controls. The distribution of genotypes in alleles, Hardy-Weinberg equilibrium, and genetic association were analyzed using the PLINK software. The linkage of disequilibrium and haplotype analyses were performed using the Haploview software.

**Results:**

Out of the 20 loci analyzed, *CYP2C19*-rs4986893, *ABCB1*-rs1045642, and *SCN2A*-rs17183814 passed Bonferroni correction; their statistical powers were > 55%. The minor allele frequencies (MAF) of *CYP2C19*-rs4986893 in the MDD group (0.0547) and BPD group (0.0533) were higher than that of the control group (0.0259, *P* < 0.05), leading to the odds ratios (ORs) of MDD (2.178) and BPD (2.122), respectively. In contrast, the lower MAFs of *ABCB1*-rs1045642 were observed in both MDD (0.3599, OR = 0.726) and BPD (0.3700, OR = 0.758) groups than controls (0.4364, *P* < 0.05). The MDD group had a higher MAF of *SCN2A*-rs17183814 than controls (0.1743 vs. 0.1207, OR = 1.538, *P* < 0.05). Moreover, a G-A haplotype composed by *CYP2C19*-rs4986893 and -rs4244285 was associated with BPD (OR = 1.361, *P* < 0.01), and the A-G haplotype increased the risks to both MDD (OR = 2.306, *P* < 0.01) and BPD (OR = 2.332, *P* < 0.001). The *CYP2C19* intermediate metabolizer and poor metabolizer (IM&PM) status was related to the raised risk of both MDD (OR = 1.547, *P* < 0.01) and BPD (OR = 1.808, *P* < 0.001).

**Conclusion:**

Our data indicate that the impaired *CYP2C19* metabolism caused by the haplotypes integrated by *CYP2C19* alleles might confer the risk to MDD and BPD, whereas the *ABCB1*-rs1045642 T allele serves as a protective factor.

## Background

Major depressive disorder (MDD) and bipolar disorder (BPD) are chronic and recurrent mood disorders affecting approximately 13% of the world’s population [1, 2]. Due to the substantial increase in healthcare expenditure and increasing suicide rate from MDD and BPD, these mood disorders form a tremendous socioeconomic burden on families and society. MDD is characterized by significant and persistent depressed mood, waned interest, slowed thinking, and cognitive impairment [3], whereas BPD is characterized by extreme mood state swing between mania and depression [4]. Previous studies have suggested that these two mood disorders do not only have overlapping symptoms but also share mechanisms [5], including metabolic dysregulation, insulin resistance, immune disorders, and neural signal transduction pathway malfunction. Several genes closely related to pathological mechanisms have been identified in previous studies. For example, polymorphisms of *CYP2C19*, *CYP2C9*, *NAT2*, *UGT1A9*, and *ABCB1* related to the activation or detoxification of drugs and endogenous substances have emerged as major genetic factors in several psychiatric disorders [6–8].

Since genetic factors with accumulative multiple variants clearly play a critical role in the etiology and pathology of polygenic mood disorders [9], characterization of the genetic features involved in etiological mechanism is particularly required. However, the potential genetic associations remain unclear, and the results of genome-wide association studies (GWAS) on mood disorders are rarely repeatable [9]. Furthermore, several studies failed to identify gene-disease correlations in patients with mood disorders [10, 11]. One probable reason for the unsuccessful generation of repeatable results to demonstrate the main effects of these genes on these diseases is that allelic frequencies may vary in different racial and ethnic backgrounds. The results from previous studies on particular genetic backgrounds cannot be applied to other populations. In this study, we investigated the candidate genes in the Han Chinese population. An effective method was developed to simultaneously analyze the pathogenic effect of these specific genes for constructing a custom single nucleotide polymorphism (SNP) detection package covering loci selected based on current assumptions and proofs from previous studies. Twenty loci from *LEPR*, *SCN2A*, *SCN1A*, *UGT1A9*, *GSK3B*, *HLA-B*, *ABCB1*, *NAT2*, *CYP2C19*, *CYP2C9*, *ANKK1*, *SH2B1*, and *INSR* were present in the SNP detection array.

In terms of the study on genetic risk could be pathogenesis support and diagnostic reference for psychiatric diseases, this study verifies the association between SNPs in 13 candidate genes and the risk of mood disorders, including MDD and BPD, in the Han Chinese population via MALDI-TOF mass spectrometry. Additionally, the effect of haplotypes and metabolism statuses were analyzed.

## Materials and methods

### Study participants

All participants were Han Chinese living in Guangdong Province, Southern China. The case group included 439 patients with MDD (158 males and 281 females) and 600 patients with BPD (258 males and 342 females) hospitalized at the Affiliated Brain Hospital of Guangzhou Medical University from February 2020 to September 2021. The diagnosis for each patient was strictly based on the DSM-V criteria [12, 13] for MDD and BPD, and was agreed by at least two independent and experienced psychiatrists. Patients were excluded if they were diagnosed with primary or comorbid physical diseases or other mental illnesses, such as schizoaffective disorder, schizophrenia, dementia, alcohol or drug addiction, post-traumatic stress disorder, obsessive-compulsive disorder, panic disorder, and anxiety disorder. The control group consisted of 464 adults (196 males and 268 females) who underwent annual physical examinations, and those with personal or family history of major psychiatric disorders were excluded. The corresponding mean ages of the control, MDD, and BPD groups were 30.7 ± 12.6 years, 29.8 ± 14.9 years, and 30.5 ± 14.7 years, respectively. Age and gender were matched between the case and control groups (*P* > 0.05). Demographic and clinical data of MDD and BPD cases are listed in Table 1.

**Table 1:** Demographic and clinical data in all groups

Characteristic	Control (*N* = 464)	MDD (*N* = 439)	BPD (*N* = 600)	*χ*^*2*^ or *F*	*P* value
Age (Mean ± SD)	30.7 ± 12.6	29.8 ± 14.9	30.5 ± 14.7	*F* = 0.431	0.650
Gender (N (%) female)	268 (57.8)	281 (64.0)	342 (57.0)	*χ*^*2*^ = 5.804	0.055
Number of hospitalizations (Mean ± SD)	----	1.5 ± 1.4	1.9 ± 1.8	----	----
Family history of mental illness (N (%))	----	72 (16.4)	122 (20.3)	----	----
Age at onset (Mean ± SD)	----	22.1 ± 13.6	23.1 ± 11.4	----	----
Education experience (N (%))				*F* = 5.630	0.466
Primary school	33 (7.1)	35 (8.0)	44 (7.3)		
Junior school	134 (28.9)	124 (28.2)	146 (24.3)		
Senior school	130 (28.0)	135 (30.8)	177 (29.5)		
College/university	167 (36.0)	145 (33.0)	233 (38.8)		
Marriage status (N (%))				*χ*^*2*^ = 0.964	0.617
Married	148 (31.9)	131 (29.8)	175 (29.2)		
Single	316 (68.1)	308 (70.2)	425 (70.8)		
Employment status (N (%))				*χ*^*2*^ = 3.512	0.173
Employed	205 (44.2)	167 (38.0)	248 (41.3)		
Unemployed/Retired	259 (55.8)	272 (62.0)	352 (58.7)		

*MDD* Major depressive disorder, *BPD* Bipolar disorder

### DNA selection

Based on previous studies on risk variants contributing to psychiatry disorders, 13 genes containing 20 SNPs were selected for further analysis. *CYP2C19* (rs12248560, rs4986893, rs4244285) was obtained from the findings of a 4-week prospective study by Strumila et al. [7]. *CYP2C9* (rs1057910) was chosen from a case-control study in a European population [14–16]. *NAT2* (rs1041983, rs1801280, rs1799929, rs1799930, rs1208) was obtained from a feature review [6]. *UGT1A9* (rs2741049) was selected based on a study by Cecil et al. [17]. *ABCB1* (rs1045642) was chosen based on mice knockout and genetic association studies [8, 18]. *LEPR* (rs1137101), *INSR* (rs2396185), *SH2B1* (rs3888190), and *GSK3B* were the candidate genes because of their involvement in the insulin resistance process [19–25]. *SCN1A* (rs2298771, rs3812718) and *SCN2A* (rs17183814) were selected due to their relation to the role of the signaling pathway in emotional disorders [26, 27]. Since *ANKK1* (rs1800497) is involved in dopaminergic pathway regulation, it might be a risk variant of MDD and BPD [28]. Since immune reaction is shaped by diverse human leukocyte antigen loci to some extent, *HLA-B* (rs2442736) is postulated to be a genetic risk factor for mood disorders [29].

### DNA extraction and SNP genotyping

EDTA-K_2_ anticoagulant blood, 2 mL, was collected from all participants for SNP detection. DNA was extracted from 0.5 mL of blood using the Blood Genomic DNA Isolation Kit (Shanghai BaiO Technology Co. Ltd), following the manufacturer’s manual. The samples were kept at − 80℃ until further analysis.

DNA samples were diluted to 5 ng/uL and then used for amplification. After the multiplex PCRs were performed, the products were treated with shrimp alkaline phosphatase to remove excess dNTPs and used as templates for the primer extension reactions using iPLEX mixture. The final products were automatically spotted on the MassARRAY SpectroCHIP. The target panels were inserted into the MALDI-TOF mass spectrometer, and SNP data were auto-analyzed by this instrument. Shanghai Kangli Medical Research Institute assisted with SNP genotyping. Twenty loci from *LEPR*, *SCN2A*, *SCN1A*, *UGT1A9*, *GSK3B*, *HLA-B*, *ABCB1*, *NAT2*, *CYP2C19*, *CYP2C9*, *ANKK1*, *SH2B1*, and *INSR* genes were typed.

### Statistical analysis

Age difference was compared using the student’s t-test, and gender and haplotype were analyzed with Pearson’s Chi-square test using the IBM SPSS (IBM, Armonk, NY) version 20. Hardy-Weinberg equilibrium analysis, genotype and allele frequencies, and association tests were conducted using the PLINK software version 1.9 (https://www.cog-genomics.org/plink) [30]. Exact test was used for Hardy-Weinberg equilibrium analysis in PLINK software. The linkage disequilibrium and haplotype analysis were performed using the Haploview software (Broad, Cambridge, MA) version 4.2 [31]. The *P* values of alleles were corrected by Bonferroni correction, in which the adjusted *P* values acquired were multiplied by SNP amount. Statistical power was calculated using the PS program on line (https://statcomp2.app.vumc.org/ps/).

## Results

### Hardy-Weinberg equilibrium analysis of 20 SNPs in all groups

Hardy-Weinberg equilibrium of 20 SNPs was tested (Table 2). The SNPs passed the Hardy-Weinberg equilibrium test in all groups, showed that sample sets were representative of the population. The GRCh38 human reference genome was used for genetic variant location. ID number and position of SNPs are shown in Table 2.

**Table 2:** Hardy-Weinberg equilibrium analysis of 20 SNPs in all groups

Gene	SNP ID	Position	*P* value		
			Control (*N* = 464)	MDD (*N* = 439)	BPD (*N* = 600)
*LEPR*	rs1137101	chr1:65592830	0.330	0.999	0.430
*SCN2A*	rs17183814	chr2:165295879	0.829	0.510	0.999
*SCN1A*	rs2298771	chr2:166036278	0.768	0.188	0.803
*SCN1A*	rs3812718	chr2:166053034	0.699	0.620	0.307
*UGT1A9*	rs2741049	chr2:233673188	0.509	0.082	0.412
*GSK3B*	rs334558	chr3:120094435	0.999	0.999	0.728
*HLA-B*	rs2442736	chr6:31378844	0.615	0.999	0.642
*ABCB1*	rs1045642	chr7:87509329	0.059	0.605	0.335
*NAT2*	rs1041983	chr8:18400285	0.707	0.209	0.805
*NAT2*	rs1801280	chr8:18400344	0.065	0.606	0.241
*NAT2*	rs1799929	chr8:18400484	0.144	0.063	0.257
*NAT2*	rs1799930	chr8:18400593	0.526	0.224	0.217
*NAT2*	rs1208	chr8:18400806	0.354	0.239	0.257
*CYP2C19*	rs12248560	chr10:94761900	0.999	0.999	0.999
*CYP2C19*	rs4986893	chr10:94780653	0.999	0.373	0.402
*CYP2C19*	rs4244285	chr10:94781859	0.123	0.648	0.999
*CYP2C9*	rs1057910	chr10:94981296	0.999	0.999	0.999
*ANKK1*	rs1800497	chr11:113400106	0.104	0.377	0.867
*SH2B1*	rs3888190	chr16:28878165	0.188	0.156	0.082
*INSR*	rs2396185	chr19:7246650	0.054	0.545	0.576

The GRCh38 human reference genome was used for genetic variant location

### Association analysis of genetic predisposition in MDD and BPD

The genotype distribution and minor allele frequencies (MAF) of each SNP are listed in Table 3. After Bonferroni correction, only *CYP2C19*-rs4986893, *ABCB1*-rs1045642, and *SCN2A*-rs17183814 were passed for subsequent analysis; their statistical powers were greater than 55%. The MAF of *CYP2C19*-rs4986893 in the MDD group (0.0547) and BPD group (0.0533) were higher than that of the control group (0.0259, *P* < 0.05). With the control group as reference, participants with the *CYP2C19*-rs4986893 A allele had odds ratios (ORs) of 2.178 and 2.122 for MDD and BPD, respectively. In contrast, both MDD (0.3599) and BPD (0.3700) groups had lower MAFs of *ABCB1*-rs1045642 than the control (0.4364, *P* < 0.05) group. Therefore, participants with the *ABCB1*-rs1045642 T allele had ORs of 0.726 and 0.758 for MDD and BPD, respectively. The MAF of the *SCN2A*-rs17183814 in patients with MDD (0.1743) was higher than that of the controls (0.1207, *P* < 0.05). Participants with the *SCN2A*-rs17183814 A allele had a 1.538-fold greater risk to suffer from MDD than those without it.

**Table 3:** Genotype distribution of SNPs in all groups

Gene	SNP ID		Genotype distribution			MAF	χ2	*P*	OR	95%CI	Statistical power
*LEPR*	rs1137101		AA	AG	GG						
		Control	11	105	348	0.1369					
		MDD	5	86	348	0.1093	3.156	0.076	0.774	0.584–1.027	28%
		BPD	10	120	470	0.1167	1.943	0.163	0.833	0.644–1.077	18%
*SCN2A*	rs17183814		GG	AG	AA						
		Control	359	98	7	0.1207					
		MDD	297	131	11	0.1743	10.34	0.001	1.538	1.181–2.001	62%
		BPD	416	168	16	0.1667	8.841	0.003	1.457	1.136–1.869	56%
*SCN1A*	rs2298771		AA	AG	GG						
		Control	387	73	4	0.0873					
		MDD	357	75	7	0.1014	1.049	0.306	1.18	0.860–1.618	10%
		BPD	498	97	5	0.0892	0.023	0.879	1.024	0.757–1.385	12%
*SCN1A*	rs3812718		GG	AG	AA						
		Control	72	228	164	0.4009					
		MDD	74	206	159	0.4032	0.01	0.92	1.01	0.837–1.219	1%
		BPD	100	275	225	0.3958	0.055	0.814	0.979	0.822–1.166	5%
*UGT1A9*	rs2741049		TT	CT	CC						
		Control	152	221	91	0.4343					
		MDD	149	198	92	0.4351	0.001	0.972	1.003	0.833–1.209	5%
		BPD	167	309	124	0.4642	1.889	0.169	1.128	0.950–1.341	16%
*GSK3B*	rs334558		TT	CT	CC						
		Control	56	212	196	0.3491					
		MDD	54	200	185	0.3508	0.005	0.941	1.007	0.830–1.222	5%
		BPD	83	287	230	0.3775	1.816	0.178	1.13	0.946–1.351	15%
*HLA-B*	rs2442736		GG	GC	CC						
		Control	422	42	0	0.0453					
		MDD	385	53	1	0.0626	2.682	0.102	1.41	0.933–2.130	23%
		BPD	544	54	2	0.0483	0.111	0.74	1.071	0.713–1.609	6%
*ABCB1*	rs1045642		CC	CT	TT						
		Control	137	249	78	0.4364					
		MDD	177	208	54	0.3599	11.01	0.001	0.726	0.601–0.877	67%
		BPD	244	268	88	0.3700	9.628	0.002	0.758	0.637–0.903	61%
*NAT2*	rs1041983		CC	CT	TT						
		Control	144	225	95	0.4472					
		MDD	130	230	79	0.4419	0.051	0.821	0.979	0.813–1.179	5%
		BPD	182	300	118	0.4467	0.001	0.981	0.998	0.840–1.186	5%
*NAT2*	rs1801280		TT	CT	CC						
		Control	416	40	8	0.0603					
		MDD	400	38	1	0.0456	1.96	0.162	0.743	0.490–1.127	17%
		BPD	554	44	2	0.0400	4.66	0.031	0.649	0.437–0.964	33%
*NAT2*	rs1799929		CC	CT	TT						
		Control	430	32	2	0.0388					
		MDD	390	45	4	0.0604	4.481	0.034	1.592	1.032–2.456	33%
		BPD	553	45	2	0.0408	0.057	0.812	1.055	0.680–1.636	36%
*NAT2*	rs1799930		GG	AG	AA						
		Control	264	176	24	0.2414					
		MDD	231	182	26	0.2665	1.506	0.22	1.142	0.924–1.412	14%
		BPD	312	250	38	0.2717	2.505	0.114	1.172	0.963–1.427	20%
*NAT2*	rs1208		AA	AG	GG						
		Control	416	44	4	0.0560					
		MDD	400	37	2	0.0467	0.805	0.37	0.825	0.542–1.256	11%
		BPD	553	45	2	0.0408	2.675	0.102	0.717	0.481–1.070	27%
*CYP2C19*	rs12248560		CC	CT	TT						
		Control	460	4	0	0.0043					
		MDD	434	5	0	0.0057	0.174	0.676	1.323	0.354–4.943	----
		BPD	594	6	0	0.0050	0.053	0.818	1.161	0.327–4.125	----
*CYP2C19*	rs4986893		GG	AG	AA						
		Control	440	24	0	0.0259					
		MDD	393	44	2	0.0547	9.781	0.002	2.178	1.323–3.588	66%
		BPD	536	64	0	0.0533	9.962	0.002	2.122	1.317–3.420	67%
*CYP2C19*	rs4244285		GG	AG	AA						
		Control	256	168	40	0.2672					
		MDD	219	179	41	0.2973	2.009	0.156	1.16	0.945–1.424	17%
		BPD	275	263	62	0.3225	7.633	0.006	1.305	1.080–1.577	50%
*CYP2C9*	rs1057910		AA	AC	CC						
		Control	433	31	0	0.0334					
		MDD	406	33	0	0.0376	0.231	0.631	1.13	0.686–1.862	6%
		BPD	568	32	0	0.0267	0.827	0.363	0.793	0.480–1.309	10%
*ANKK1*	rs1800497		GG	AG	AA						
		Control	168	208	88	0.4138					
		MDD	143	224	72	0.4191	0.053	0.818	1.022	0.848–1.233	5%
		BPD	202	290	108	0.4217	0.133	0.715	1.033	0.868–1.229	6%
*SH2B1*	rs3888190		CC	AC	AA						
		Control	396	63	5	0.0787					
		MDD	365	68	6	0.0911	0.902	0.342	1.174	0.843–1.636	10%
		BPD	498	93	9	0.0925	1.268	0.26	1.194	0.877–1.625	12%
*INSR*	rs2396185		AA	AC	CC						
		Control	336	124	4	0.1422					
		MDD	328	101	10	0.1378	0.073	0.786	0.964	0.739–1.258	5%
		BPD	408	171	21	0.1775	4.789	0.029	1.301	1.027–1.648	33%

χ^2^, *P*, OR, and 95%CI in this table indicate chi-square value, *P* value, odds ratio, and 95% confidence interval of minor alleles, respectively

*MAF* Minor allele frequency

### Linkage disequilibrium analysis and haplotype analysis

**Fig. 1:**
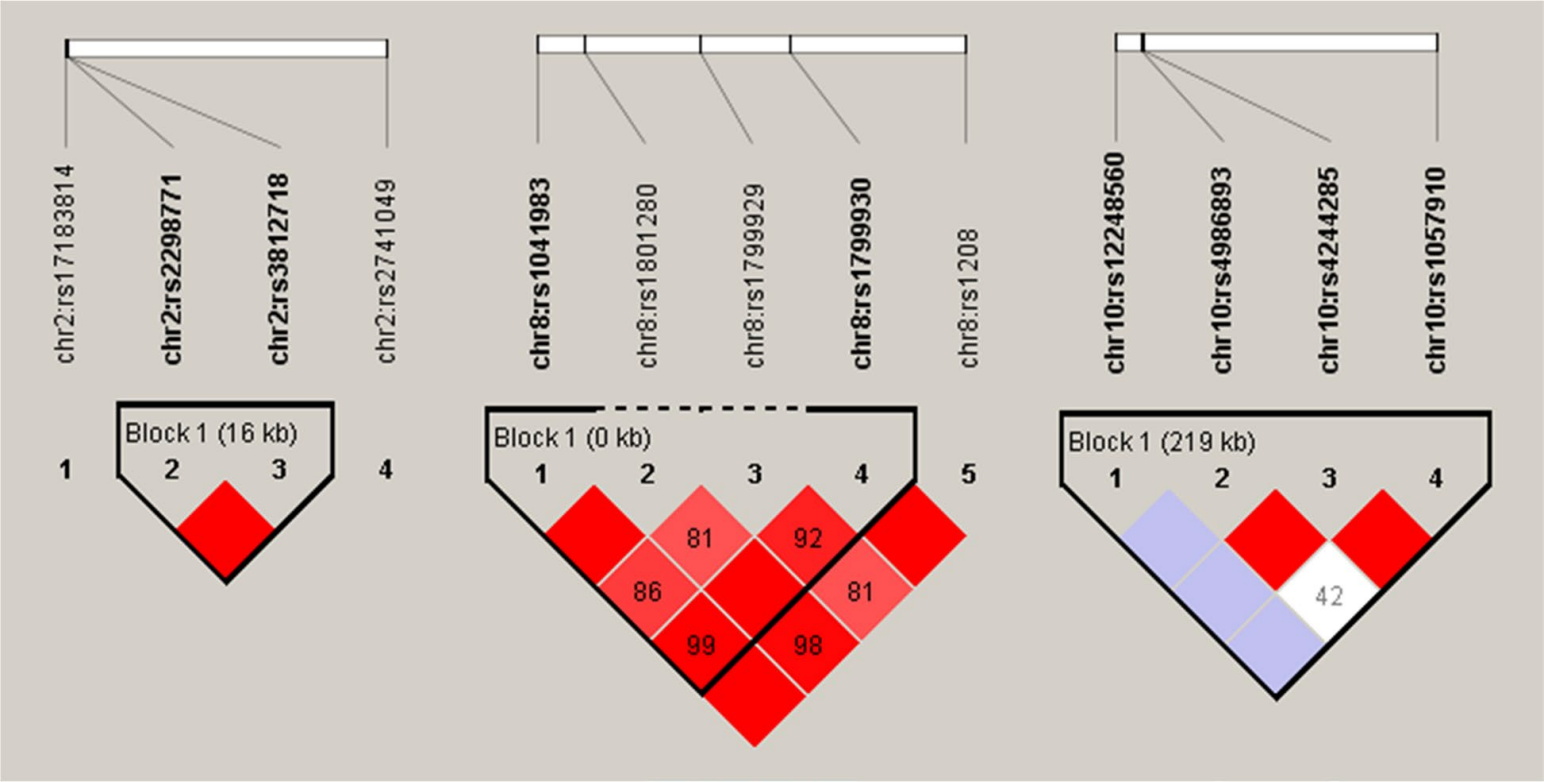
Linkage disequilibrium plots of cases and controls. White signifies D‘<1, LOD < 2; blue signifies D’=1, LOD < 2; shade of pink/red signifies D‘<1, LOD ≥ 2; bright red signifies D’= 1, LOD ≥ 2

**Fig. 2:**
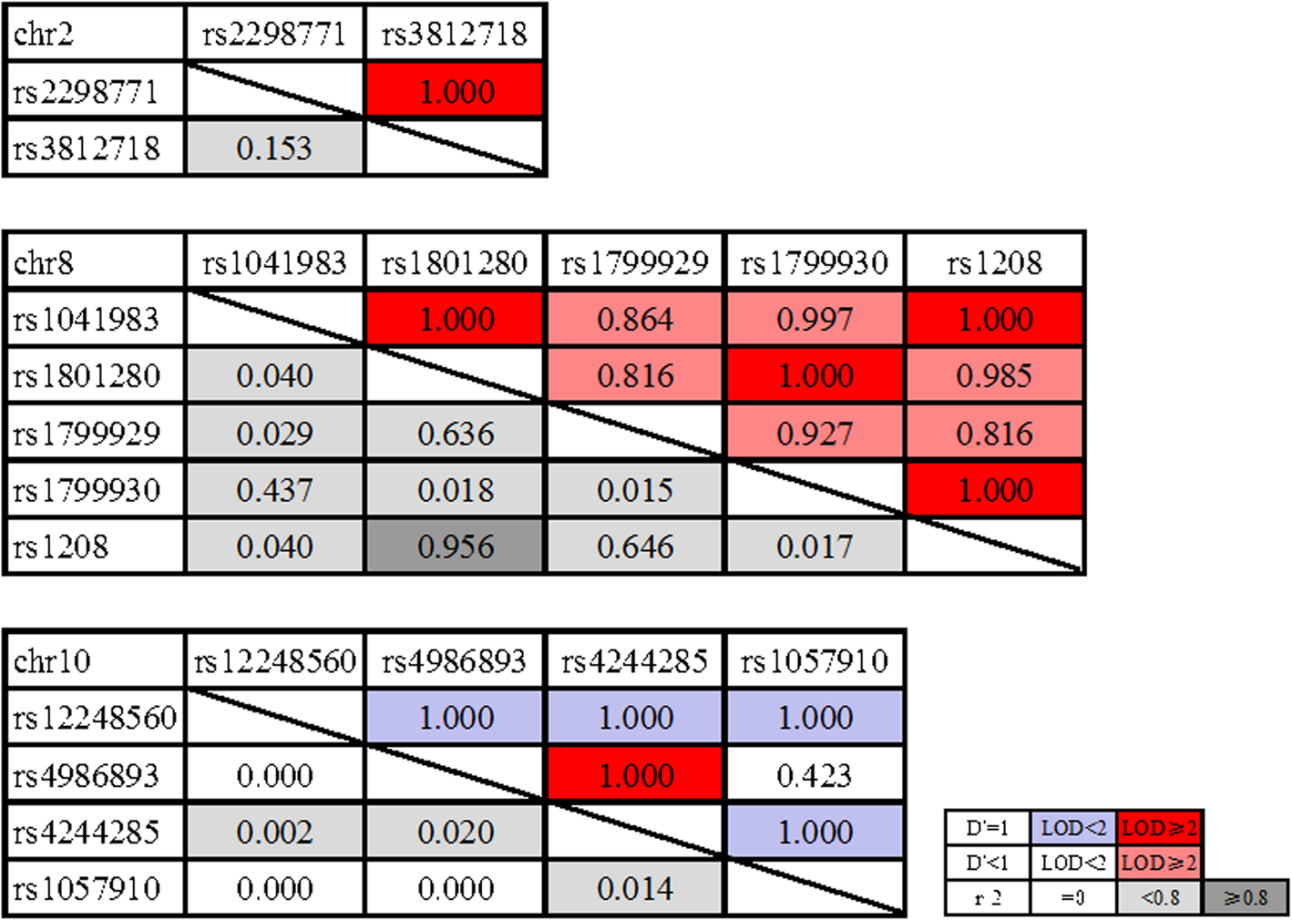
Pairwise linkage disequilibrium status in chromosome 2, chromosome 8, and chromosome 10. D’ and r^2^ are indicated in upper and lower triangles, respectively. In addition, the color codes in the lower right corner show the strength of linkage disequilibrium status of SNPs. For the upper triangles, white signifies D‘<1, LOD < 2; blue signifies D’=1, LOD < 2; shade of pink/red signifies D‘<1, LOD ≥ 2; bright red signifies D’=1, LOD ≥ 2. For the lower triangles, white means r^2^ = 0, light grey means r^2^ < 0.8, and dark grey means r^2^ ≥ 0.8

Among the 20 SNPs, 13 SNPs were present in three chromosome (chr) blocks, including chr 2 (SCN2A-rs17183814, SCN1A-rs2298771, SCN1A-rs3812718, and UGT1A9-rs2741049), chr 8 (NAT2-rs1041983, -rs1801280, -rs1799929, -rs1799930, and -rs1208), and chr 10 (CYP2C19-rs12248560, -rs4986893, -rs4244285 and CYP2C9-rs1057910) (Fig. 1). The significant SNP (rs4986893) was located at the block in chr 10, and showed strong linkage disequilibrium with rs4244285 (Fig. 2). Therefore, the haplotypes of rs4986893 and rs4244285 were reconstructed, and a total of three haplotypes were observed in the studied population. The distributions of these haplotypes were significantly different between cases and controls. Moreover, the ORs of the haplotypes between the case and control groups were analyzed (Table 4). The G-A haplotype from rs4986893 and rs4244285 was related to the increased risk of BPD (χ2 = 10.068, OR = 1.361, *P* = 0.002), while the A-G haplotype raised both the risks of MDD and BPD (χ^2^ = 11.145, OR = 2.306, P = 0.001; χ^2^ = 12.549, OR = 2.332, *P* < 0.001).

**Table 4:** Haplotype analysis of rs4986893 and rs4244285

	rs4986893	rs4244285		Control(*n* = 464)	MDD(*n* = 439)	BPD(*n* = 600)
Haplotype 1	G	G		656	569	750
Haplotype 2	G	A		248	261	386
Haplotype 3	A	G		24	48	64
	MDD			BPD		
	χ2	*P*	OR (CI 95%)	χ2	*P*	OR (95% CI)
Overall	13.137	0.001	––	20.065	< 0.001	––
2 vs. 1	3.359	0.067	1.213 (0.987, 1.492)	10.068	0.002	1.361(1.125, 1.648)
3 vs. 1	11.145	0.001	2.306(1.395, 3.812)	12.549	< 0.001	2.332 (1.442, 3.772)

### *CYP2C19* metabolizer status distribution in controls and cases

When genotypes composed of rs4244285 and rs4986893 were translated into predicted CYP2C19 metabolism, it could be categorized as normal metabolizer (NM), intermediate metabolizer (IM), poor metabolizer (PM). NM was the subject carried none of these defective alleles, while IM was the subject had one defective allele and PM was the one had two defective alleles. The distributions of these CYP2C19 metabolizer statuses were significantly different between cases and controls. The frequencies of IM&PM status were higher in MDD (57.40%, OR = 1.547) and BPD (61.17%, OR = 1.808) cases than those in controls (46.55%, *P* < 0.05), showed in Table 5.

**Table 5:** *CYP2C19* metabolizer status distribution in controls and cases with MDD and BPD

	Control(*n* = 464)	MDD(*n* = 439)	BPD(*n* = 600)			
NM	248 (53.45%)	187 (42.60%)	233 (38.83%)			
IM	160 (34.48%)	195 (44.42%)	283 (47.17%)			
PM	56 (12.07%)	57 (12.98%)	84 (14.00%)			
IM&PM	216 (46.55%)	252 (57.40%)	367 (61.17%)			
	MDD			BPD		
	χ2	*P*	OR (CI 95%)	χ2	*P*	OR (95% CI)
NM vs. IM vs. PM	11.330	0.003	––	23.215	< 0.001	––
IM&PM vs. NM	10.639	0.001	1.547(1.190,2.012)	22.563	< 0.001	1.808(1.415,2.311)

*NM* Normal metabolizer, *IM* Intermediate metabolizer, *PM* Poor metabolizer, *IM&PM* Intermediate metabolizers plus poor metabolizers

## Discussion

Many etiopathogenetic mechanisms are involved in mood disorders, such as MDD and BPD. Due to the common symptoms and shared etiologies between these disorders, we sought to clarify whether correlations existed between candidate genetic variants in selected genes and susceptibility to MDD and BPD in the Han Chinese population. To the best of our knowledge, this study is the first Chinese study to examine the implication of 13 genes on both MDD and BPD risk, covering *LEPR*, *SCN2A*, *SCN1A*, *UGT1A9*, *GSK3B*, *HLA-B*, *ABCB1*, *NAT2*, *CYP2C19*, *CYP2C9*, *ANKK1*, *SH2B1*, and *INSR*. Our data suggested that *SCN2A*-rs17183814, *ABCB1*-rs1045642, and *CYP2C19*-rs4986893 had associations with MDD or BPD, providing evidence for genetic vulnerability to mood disorders, and provided a basis for understanding the etiology of these disorders for earlier prevention.

The neuronal voltage-gated sodium channel, which modulates neuron excitability and initial transduction, is encoded by the *SCN2A* gene expressed in the initial segment of the axon and plays a crucial part in neuronal pathfinding and neurite outgrowth [32]. Once neuronal voltage-gated sodium channels are deficient in mature neurons, action potential is back-propagated, dendrite excitability is reduced, and synaptic efficacy is damaged [33]. Diminished channel function interferes with the neural signal transduction pathway, resulting in the occurrence of MDD, BPD, and autism spectrum disorder [26, 34]. Our data could not confirm the association between SCN2A-rs17183814 and BPD in European and Chinese Han populations [26]. We thought the reasons for the discrepancy might be due to the heterogeneity in the cases, the differences in racial composition and the smaller sample sizes than Zhao’s study (1146 BPD cases and 1956 controls). Additionally, we found that the A allele of *SCN2A*-rs17183814 increased the odds of developing MDD by 1.583-fold, which was different from the contribution of the G allele to the prevalence of MDD (OR = 1.116) observed by Zhao [26]. The biological link between the locus and affective disorders needs further clarification.

*ABCB1*, which encodes a permeability glycoprotein that is highly expressed in the brain for exporting various hydrophobic compounds, plays a vital role in forming a protective physiological barrier and emerges as an active eliminator for xenobiotics and cellular metabolites [35]. The vulnerability to MDD can be predicted with *ABCB1* by altering the activity of the hypothalamic-pituitary-adrenal axis [36]. The C allele of the *ABCB1*-rs1045642 polymorphism was connected with boosted interpersonal sensitivity among Japanese populations [8]; this allele has been generally accepted as one of the vulnerability factors for depression. Ozbey et al. [37] showed in a Turkish population that *ABCB1*-rs1045642 C allele and CC genotype were associated with susceptibility to the development of MDD. In addition, a study using a mouse model has shown that higher cortisol levels accumulated in the plasma and brain of *ABCB1* -/- knockout mice [18]. Based on these findings, we assumed that the *ABCB1*-rs1045642 C allele over-expresses the permeability glycoprotein, restricting the entry of cortisol into the brain. This leads to a lower cortisol level in the brain and higher interpersonal sensitivity. Negative feedback from the lower cortisol level can lead to a hyperactive hypothalamic-pituitary-adrenal axis, which promotes the release of cortisol and probably causes mood disorders. Consequently, T allele carriers may have lower risks of MDD and BPD. T allele carriers of three *ABCB1* loci, including rs1045642, have nearly 70% less risk of MDD among male Portuguese individuals [38]. A Chinese study showed that the TG haplotype of rs1045642–rs2032582 carriers reduced MDD risk by approximately 53% [39]. Our results were consistent with those of previous studies, where the T allele lowered the risks for MDD and BPD by 0.726- and 0.758-fold, respectively. However, the association between *ABCB1*-rs1045642 polymorphism and mood disorders has not been established. Some studies have suggested that *ABCB1*-rs1045642 T allele as the variant contributes to the predisposition of MDD [40, 41].

The CYP2C19 enzyme plays a critical role in metabolizing not only drugs or xenobiotics that affect therapeutic outcomes but also endogenous substrates containing steroid hormones, vitamin D, eicosanoids, arachidonic acids, and cholesterol that could also confer susceptibility to many diseases [42, 43]. Recent studies have suggested that impaired CYP2C19 metabolizers had higher self-rated Beck Depression Inventory-II scores than normal metabolizers. Damaged CYP2C19 enzyme activity was associated with more severe MDD, despite CYP2C19-metabolized medication treatment and treatment discrepancy status [7]. *CYP2C19* polymorphism has been demonstrated to affect the conversion and degradation of endogenous compounds, including psychoactive steroid hormones (e.g. estrone, estradiol, progesterone, and testosterone) in in vitro studies [44]. Our study findings suggest that the A allele of *CYP2C19*-rs4986893 had a 2.178-fold higher prevalence of MDD and 2.122-fold increased possibility of BPD occurrence. The haplotypes of rs4986893-G and rs4244285-A might increase the risk for BPD, while the rs4986893-A and rs4244285-G haplotype soared both the risks of MDD and BPD. Additionally, the frequencies of IM&PM status were higher in MDD and BPD cases than those in controls, which also meant defective allele (rs4986893 or rs4244285) was related to the raised risk of both MDD and BPD. Our hypothesis is that the A allele of *CYP2C19*-rs4986893, as a variant, encodes impaired CYP2C19 enzyme, promoting steroid hormone disequilibrium, and resulting in a change in hypothalamic-pituitary-adrenal axis activity and mood disorder development. The hypothesis could be verified by the G-A and A-G haplotype found in the current study, which carried genetic variant and induced impaired metabolic enzyme activity. Nevertheless, contrasting results indicated that elevated CYP2C19 expression is related to depressive symptoms [45, 46]. These deviations can be explained by inter-study discrepancies in *CYP2C19* frequency or study methods.

This study had several limitations. First, the channel function caused by *SCN2A*-rs17183814 mutation was not examined, and the substrate concentrations due to *ABCB1*-rs1045642 and *CYP2C19*-rs4986893 polymorphisms were not measured. These would have helped to characterize the physiological mechanisms. Second, the location of 20 candidate loci in various chromosomes make analysis of the effect of haplotypes on diseases difficult. Third, this study did not include controversial risk genes for depression, such as *SLC6A4* and *5-HTTLPR*; therefore, further investigations are needed [10, 14].

In conclusion, we have provided additional evidence for genetic association, confirming that the *CYP2C19*-rs4986893 A allele is a risk factor and *ABCB1*-rs1045642 T allele is protective for MDD. For the first time, we showed that these two variants have a similar effect on BPD. Additionally, the *SCN2A*-rs17183814 A allele was found to increase the morbidity of MDD. The haplotype integrated by *CYP2C19* alleles, and the *CYP2C19* metabolizer status which was categorized as IM or PM might contribute to the risk of developing mood disorder.

## Data Availability

The datasets used or analysed during the current study available from the corresponding author on reasonable request.
